# Homology modeling and molecular docking simulation of some novel imidazo[1,2-a]pyridine-3-carboxamide (IPA) series as inhibitors of *Mycobacterium tuberculosis*

**DOI:** 10.1186/s43141-020-00102-1

**Published:** 2021-01-20

**Authors:** Mustapha Abdullahi, Shola Elijah Adeniji, David Ebuka Arthur, Abdurrashid Haruna

**Affiliations:** 1 Faculty of Physical sciences, Department of Chemistry, Ahmadu Bello University, P.M.B. 1044, Kaduna State Zaria, Federal Republic of Nigeria; 2 Department of Chemistry, Baze University, Abuja, Nigeria

**Keywords:** Molecular docking, Binding affinity, Active sites, Homology, Modeling, Hydrogen bonds

## Abstract

**Background:**

Tuberculosis (TB) remains a serious global health challenge that is caused by *Mycobacterium tuberculosis* and has killed numerous people. This necessitated the urgent need for the hunt and development of more potent drugs against the fast-emerging extensively drug-resistant (XDR) and multiple-drug-resistant (MDR) *M. tuberculosis* strains. *Mycobacterium tuberculosis* cytochrome b subunit of the cytochrome bc1 complex (QcrB) was recognized as a potential drug target in *M. tuberculosis* (25618/H37Rv) for imidazo[1,2-a]pyridine-3-carboxamides whose crystal strucuture is not yet reported in the Protein Data Bank (PDB). The concept of homology modeling as a powerful and useful computational method can be applied, since the *M. tuberculosis* QcrB protein sequence data are available.

**Results:**

The homology model of QcrB protein in *M. tuberculosis* was built from the X-ray structure of QcrB in *M. smegmatis* as a template using the Swiss-Model online workspace. The modeled protein was assessed, validated, and prepared for the molecular docking simulation of 35 ligands of N-(2-phenoxy)ethyl imidazo[1,2-a] pyridine-3-carboxamide (IPA) to analyze their theoretical binding affinities and modes. The docking results showed that the binding affinity values ranged from − 6.5 to − 10.1 kcal/mol which confirms their resilience potency when compared with 6.0kcal/mol of isoniazid standard drug. However, ligands 2, 7, 22, 26, and 35 scored higher binding affinity values of − 9.60, − 9.80, − 10.10, − 10.00, and − 10.00 kcal/mol, and are respectively considered as the best ligands among others with better binding modes in the active site of the modeled QcrB protein.

**Conclusion:**

The information derived in this research revealed some potential hits and paved a route for structure-based drug discovery of new hypothetical imidazo pyridine amide analogs as anti-tubercular drug candidates.

## Background

Tuberculosis (TB) is a respiratory disease caused by the *Mycobacterium tuberculosis* organism that is one of the world’s health threats [1]. Nigeria is currently ranked 7th out of the 30 countries that are with high TB cases globally, and 2nd to be known in Africa [2]. Numerous imidazo pyridine amide (IPA) analogs were identified by high-throughput screening of chemical databases and libraries [3]. The IPA compounds were first reported as potential anti-*M. tuberculosis* candidates in 2011, and their response activity against *M. tuberculosis* showed the H37Rv strain to be within the submicromolar range [3, 4]. Q203 is an imidazo[1,2-a]pyridine-3-carboxamide candidate which is currently in clinical trials (phase II). It was reported to have improved the inhibitory response potency against XDR and MDR (TB) clinical isolates [5]. Furthermore, as novel series of anti-TB inhibitors targeting QcrB, IPAs have recently reaped immense interest; several other novel classes of new IPAs were described to have effective antimycobacterial response [5].

Cytochrome bc1 complex is crucial in the electron aerobic chain or cellular respiratory chain for transferring an electron from ubiquinol to cytochrome c across the membrane which aids in cellular activity and ATP synthesis [6]. This complex is not universal in prokaryotes but well-known in its activity. For instance, there is no bc1 complex in *Escherichia coli*. Several genetic methods have suggested that cytochrome bc1 complex is a potential molecular target in *M. tuberculosis* [7]. The cytochrome bc1 complex is made up of three (3) basic subunits namely: (i) Rieske iron-sulfur protein A subunit (QcrA), (ii) cytochrome B subunit (QcrB), and (iii) cytochrome C subunit (QcrC) [4, 8]. However, the cytochrome B subunit (QcrB) was regarded as the main actor for a functioning bc1 complex due to its coordinating action with other components of the whole bc1 complex [9]. Therefore, it can be inferred that QcrB is a promising drug target for *M. tuberculosis* based on its criticality in respiration function. Similarly, the cytochrome B subunit is identified as the target of non-selective agents like stigmatellin as well as the drug target of the atovaquone (antimalarial agent) [10]. Experimental protein structure solution by X-ray crystallography or NMR is expensive and requires intensive labor. As such, many experimental structures of proteins in any given proteome are not yet available. Until now, the cytochrome bc1 complex structure of *M. tuberculosis* has not been reported. But, the QcrB structures in numerous species (like mammals and bacteria) have been elucidated with cofactors and bound inhibitors by the technique of X-ray crystallography [6]. Also, the residual interaction between the QcrB protein subunit and its active ligands is still not clear. Comparative modeling of protein, also known as homology modeling, predicts the 3D structure of a query protein (target) sequence based on alignment of a known experimental structure of a homologous protein (template). Therefore, the development of a highly predictive binding model for active ligands could deepen the structural insight of the QcrB subunit active sites. In the present research work, a 3D structure of *M. tuberculosis* QcrB was constructed via the homology modeling method. Subsequently, the molecular docking simulation of 35 IPA ligands and the modeled *M. tuberculosis* QcrB as the target was performed in order to compute their theoretical binding affinities and explore the protein-ligand interactions of the best complexes formed.

## Methods

### Data set

**Fig. 1:**
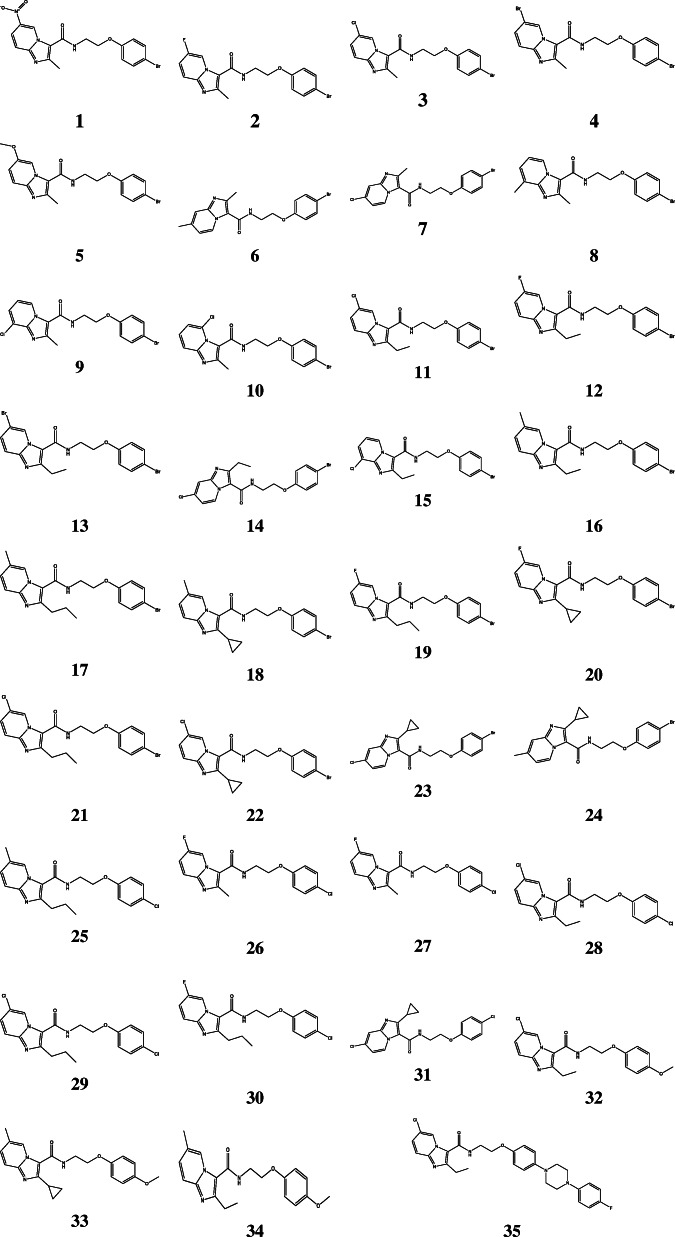
2D chemical structures of imidazo[1, 2-a] pyridine-3-carboxamides (IPAs) retrieved from Wang et al. [5]

Thirty-five (35) compounds of N-(2-phenoxy)ethyl imidazo[1,2-a] pyridine-3-carboxamides (IPAs) as active anti-tubercular agents were selected from the literature [5]. The chemical structure of each ligand was drawn accurately using ChemDraw Ultra 12.0 (Fig. 1), then further optimized with the Spartan 14 software based on density functional level of theory (DFT) at Becke’s three-parameter Lee-Yang-Parr hybrid functional basis set (B3LYP/6-31G**) in a vacuum [11].

### Homology modeling

The QcrB protein sequence for *M. tuberculosis* (strain ATCC 25618/H37Rv) was retrieved from the UniProtKB (Universal Protein Resource Knowledgebase) webserver [12], http://www.uniprot.org with accession code: P9WP37 [13]. The code was exported to SWISS-MODEL online workspace (https://www.swissmodel.expasy.org) as input for its validation. The identification of suitable templates based on Basic Local Alignment Search Tool (BLAST) [14] and a Hidden Markov model (HMM-HMM)-based lightning-fast iterative sequence search (HHblits) were all carried out to obtain the target template alignments [15]. The top-ranked aligned template from the alignment results was selected to build a new energy-minimized protein model by using the ProMod3 modeling engine [16]. The reliability of the modeled 3D structure was assessed using the Qualitative Model Energy Analysis (QMEAN) [17] and Global Model Quality Estimation (GMQE) scores [18]. The QMEAN score of below − 4.0 depicts low quality of the predicted structure, while the GMQE score ranges between 0 and 1, and a higher score corresponds to higher reliability [16]. Furthermore, structure validation of the modeled QcrB protein for stereochemical quality and Ramachandran plot were generated from the online workspace.

### Target protein and ligand preparation

**Fig. 2:**
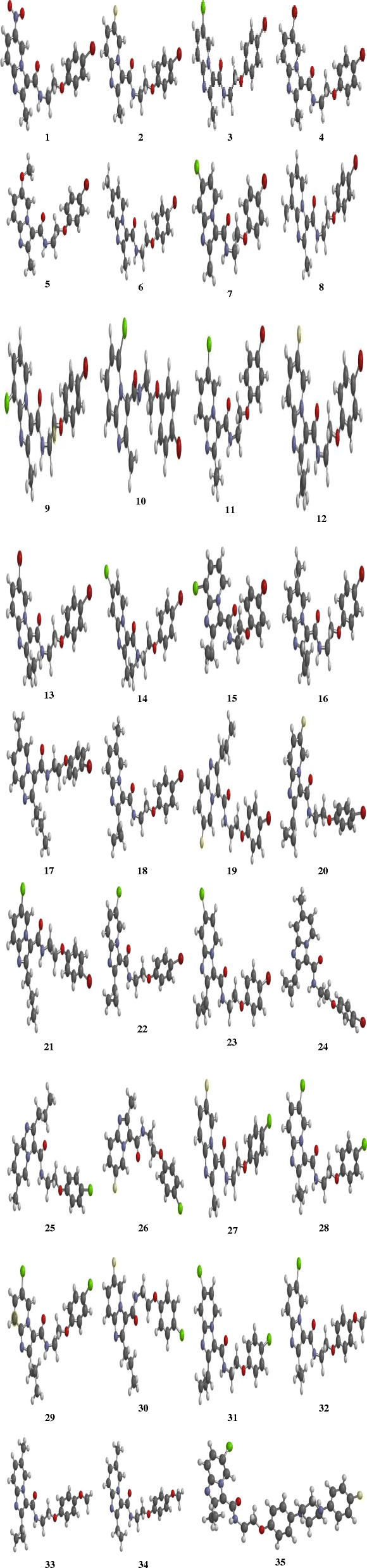
DFT-optimized chemical structures of the imidazo[1, 2-a] pyridine-3-carboxamides (IPAs)

The homology modeled QcrB target protein (.pdb format) was downloaded from SWISS-MODEL workspace [16]. The modeled protein was considered as the receptor, and the complexed ligands were manually removed using Discovery Studio. Furthermore, the docking simulation of the 35 optimized molecules (Fig. 2) was done using the PyRx virtual screening software (AutoDock Vina) [19]. Besides, the Vina wizard employs a gradient algorithm search for predicting the binding scores and modes of the ligands in the active sites of the receptors. The docking results with the highest binding score was visualized to assess the molecular interactions with the aid of the UCSF Chimera software package v1.10.1 and Discovery Studio Visualizer v16.1.0.15350 [20, 21].

## Results and discussion

### Homology modeling and structural validation

**Fig. 3:**
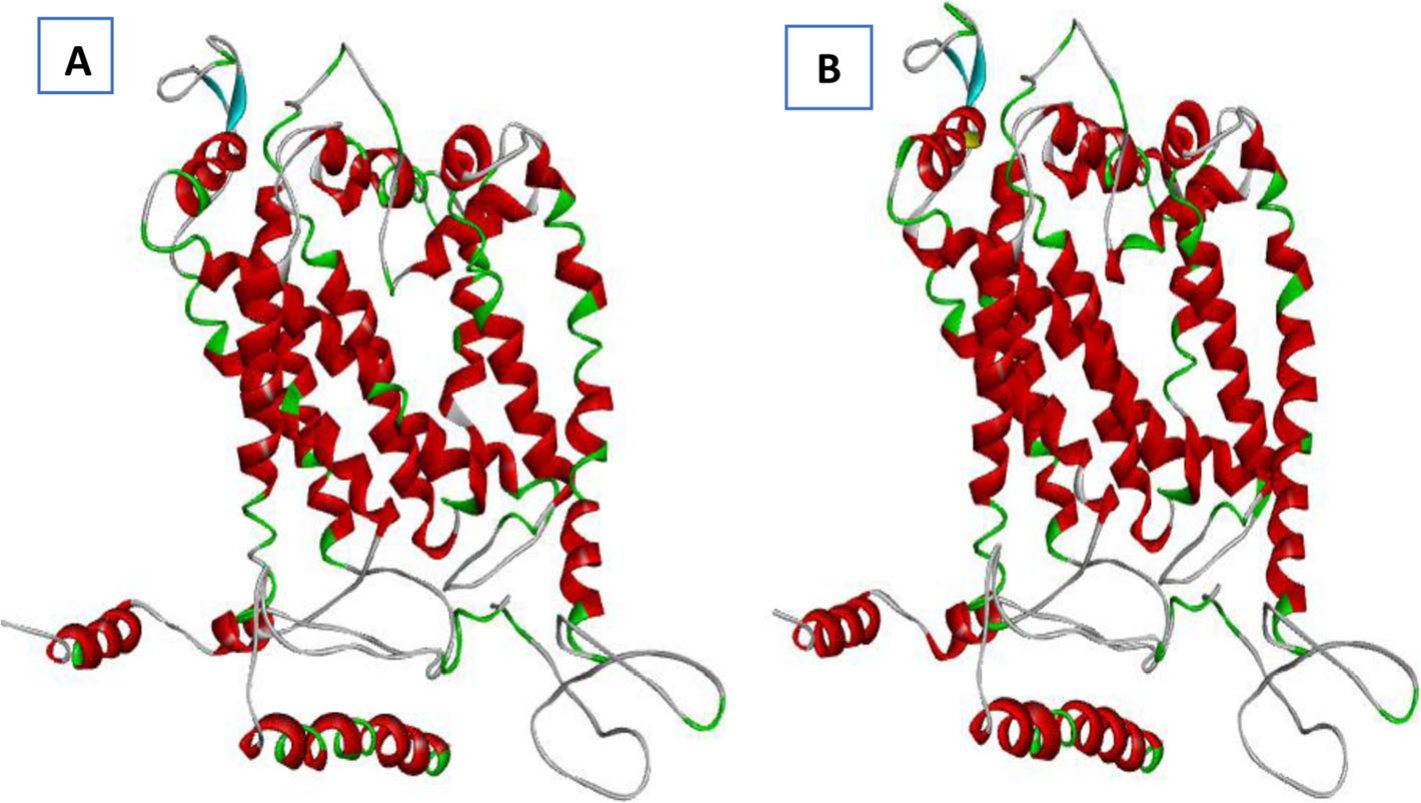
Three-dimensional (3D) structures of QcrB protein. **a** 3D structure of QcrB protein of *M. smegmatis* (PDB: 6HWH). **b** Predicted 3D structure of *M. tuberculosis* of QcrB protein

The crystal structure of cytochrome b subunit of the cytochrome bc1 complex (QcrB) in *M. tuberculosis* is not available in the Protein Data Bank database (PDB). As such, the SWISS-MODEL template library was searched with BLAST (close homologs) and HHBlits (remote homologs) as mentioned previously for evolutionary related structures matching the target sequence [22]. The results of the searching revealed a ubiquinol-cytochrome C reductase QcrB structure of a functional obligate respiratory supercomplex from *Mycobacterium smegmatis* (PDB: 6HWH) as the closest template [23], and it shares 99.5% query coverage and 82.56% identity with *Mycobacterium tuberculosis* QcrB (Fig. 3a). Therefore, the homology model of *M. tuberculosis* QcrB subunit was built with GMQE score of 0.91 and QMEAN of − 3.86 which suggests good quality and reliability (Fig. 3b). Local appraisals of model quality in terms of the QMEAN scoring function with respect to residue number and as a global score in relation to PDB structure set of high-resolution (*Z* score) was generated from the workspace.

**Fig. 4:**
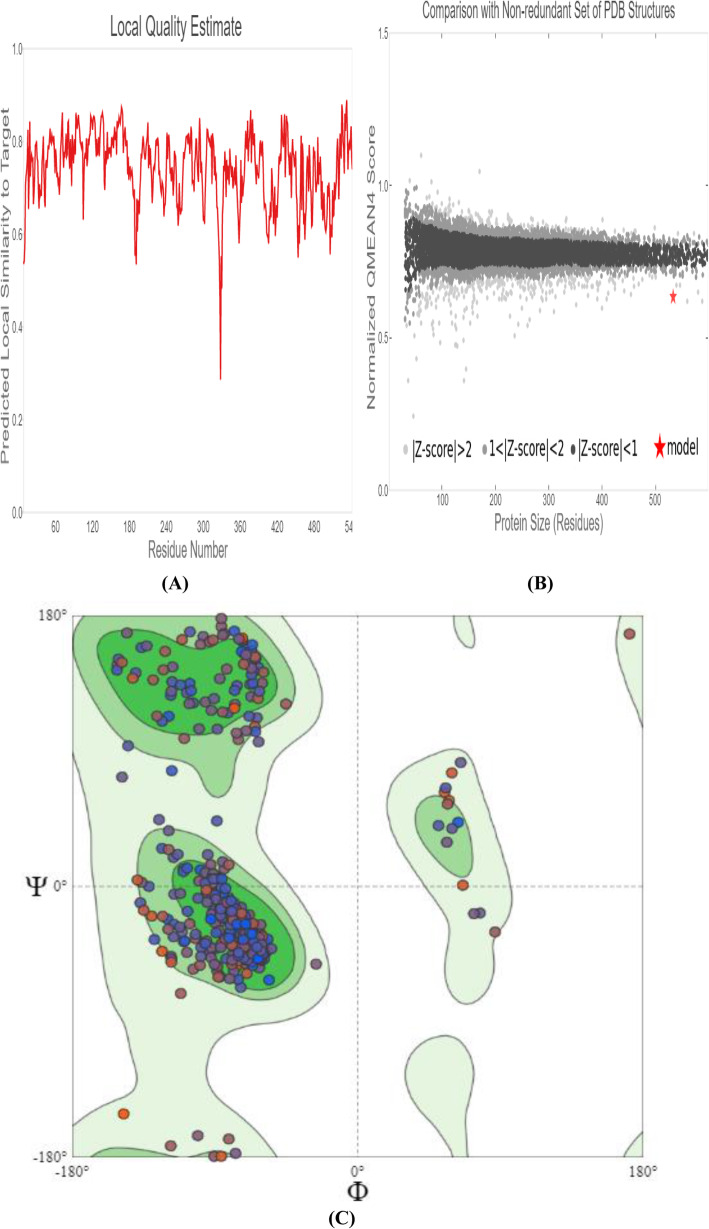
Structure validation of modeled *M. tuberculosis* QcrB protein structure: **a** Local quality estimate of the residue graph. **b** Comparison of the modeled protein structure with non-redundant set of PDB structures. **c** Ramachandran plot of the homology modeled QcrB protein for all non-glycine/proline residues

The graph of the predicted local similarity to target versus the residue number of the predicted 3D structure of the modeled protein was plotted so as to estimate the local quality of the residues (Fig. 4a). It was observed that most of the residue scores are close to 1, signifying that the predicted model has a good local quality estimate, while residues that score lower than 0.6 are said to be of low quality. Figure 4 b shows a plot of normalized QMEAN score against protein size which relates the model quality scores of an individual model to values obtained for experimental structures of similar size. The structure of the modeled protein was found within the range in comparison with the non-redundant set of PDB structures which affirms its reliability. In theory, the dihedral angle (ω) of the protein backbone is limited to the amide bond (C and N) planarity in addition to the hybridization of atomic orbitals involved which resulted in a resonance structure with a permanent dipole and double binding sites. The dihedral angle values ѱ(Psi) and ϕ(Phi) are controlled by the steric hindrance between the side-chain atoms and neighboring peptide bonds. The Ramachandran map is an efficient approach to visualize favored regions for backbone dihedral angles ѱ(Psi) against ϕ(Phi) of amino acid residues. It involves plotting the ѱ(Psi) scores on the y-axis and the ϕ(Phi) on the x-axis with angle spectrum ranging from − 180º to + 180º to predict the secondary structure and possible conformation of the peptide.

Figure 4 c shows the Ramachandran plot of the homology model of QcrB protein for all non-glycine or non-proline residues generated by the Swiss-Model workspace. The colored contour (green) represents the allowed and favored regions. However, the plot revealed 93.25% Ramachandran favored, and the most dominant amino acid residues are predicted to be contained in right-handed alpha-helices as secondary structure.

### In silico molecular docking results

The in silico molecular docking simulation hunts the best binding modes or the conformation of the ligand with the active residues of a protein target [21]. Furthermore, the assessment of docking output depends on the highly negative magnitude of binding affinity (lowest binding energy) portraying the best conformation of the ligand in the active pockets of the target. As stated earlier, the homology-modeled QcrB protein was used as the receptor to perform in silico docking computational studies on a series of thirty-five (35) IPA compounds.

**Fig. 5:**
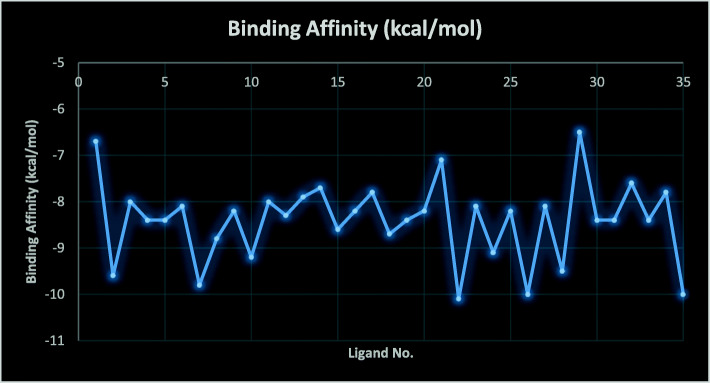
Binding affinity scores of the IPAs (ligands) with the modeled QcrB protein

Figure 5 depicted the binding affinity scores of the best ligand’s binding modes ranged from − 6.5 to − 10.1 kcal/mol which affirms their resilient potency as experimentally reported by Wang et al. [5]. In addition, the ligands were predicted to have better binding affinity values than isoniazid (standard drug) with a binding score of − 6.0 kcal/mol. Thus, ligand numbers 2, 7, 22, 26, and 35 with higher binding affinity values (− 9.60, − 9.80, − 10.10, − 10.00, − 10.00 kcal/mol) among others were selected as the best to assess their residual interactions by using the Discovery Studio software.

**Fig. 6:**
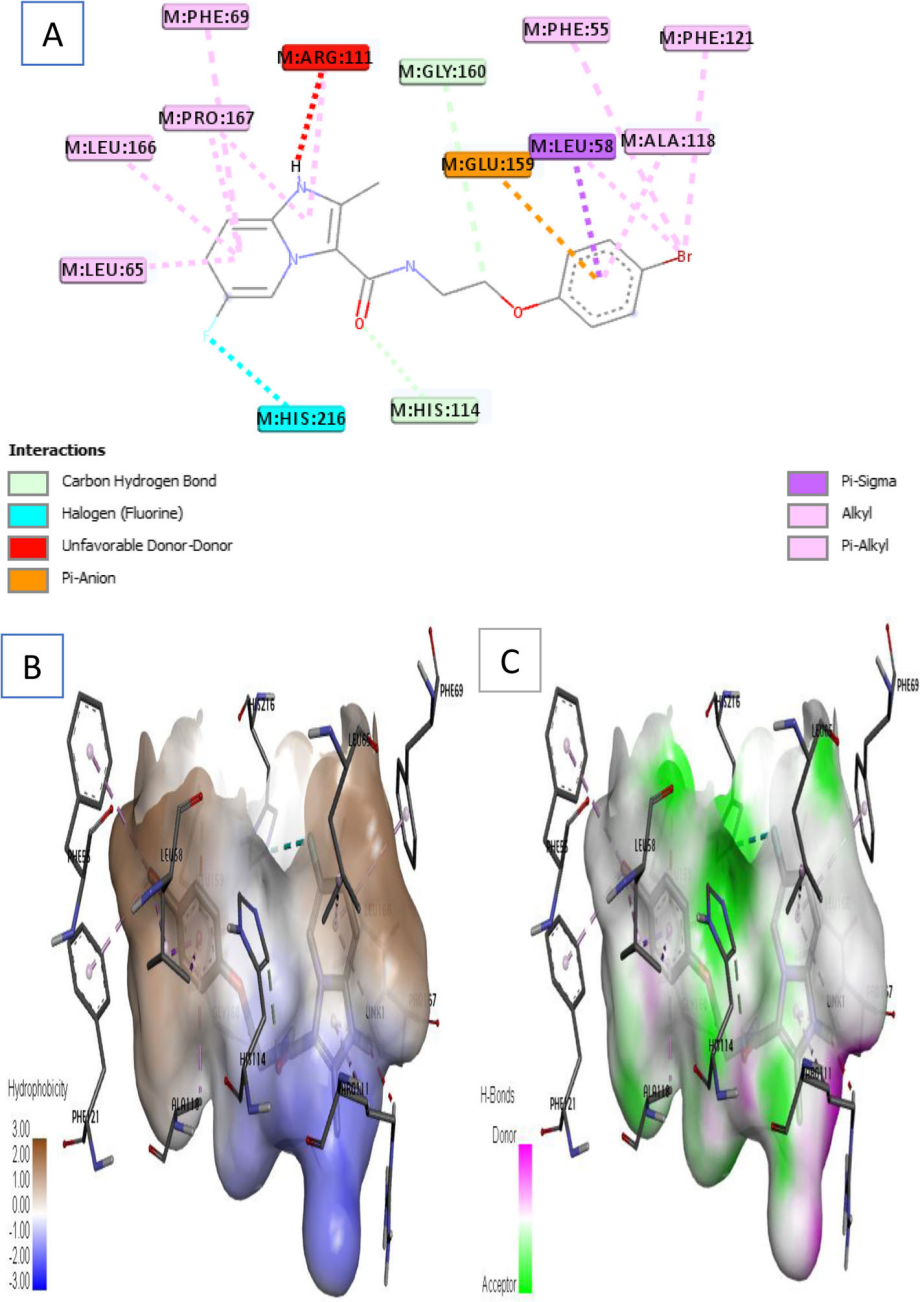
Docking interactions of complex 2. **a** 2D docked view of complex 2. **b** Hydrophobicity surfaces of complex 2. **c** Hydrogen bond interactions of complex 2

Ligand 2 formed ten (10) hydrophobic interactions with 9 amino acid residues as shown in Fig. 6a. The imidazo[1,2-a] pyridine fragment of ligand 2 formed five (5) pi-alkyl interactions with Leu 65, Leu 166, Pro 167, Phe 69, and Arg 111 at different distances, while the bromo substituent (-Br) attached to the benzene formed three (3) pi-alkyl interaction with Phe 55, Leu 58, and Phe 121 residues. Also, delocalized Π-electron of the benzene ring interacted with Leu 58 to form pi-sigma hydrophobic and pi-anion interaction with Glu 153 (electrostatic) respectively. Other noticeable interactions include C–H bond with His 216 and Gly 160, unfavorable donor-donor interaction with Arg 111, and halogen interaction between fluoro substituent of imidazo[1,2-a] pyridine fragment and His 216 accordingly.

**Fig. 7:**
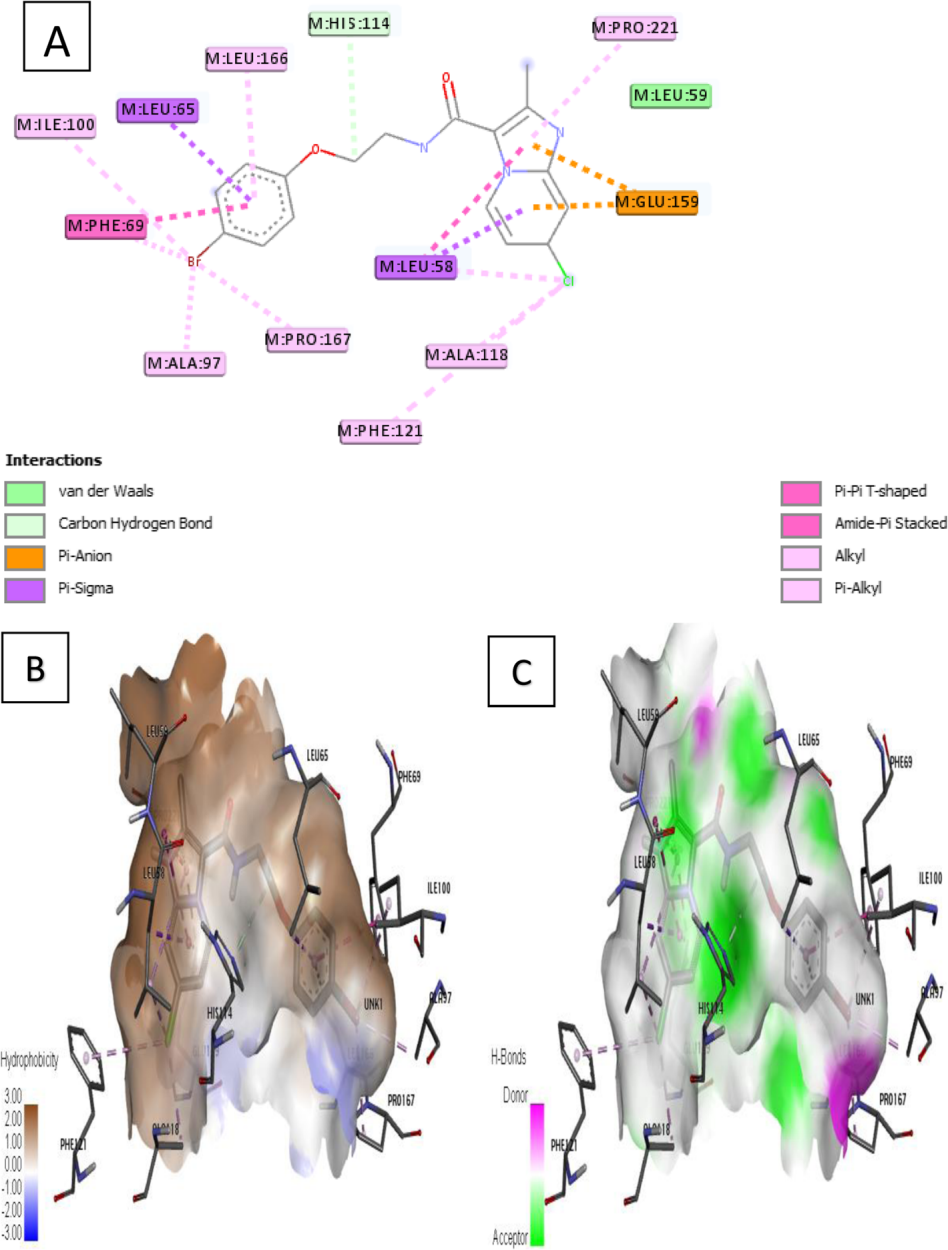
Docking interaction of complex 7. **a** 2D docked view of complex 7. **b** Hydrophobicity surfaces of complex 7. **c** Hydrogen bond surfaces of complex 7

Ligand 7 formed thirteen (13) major hydrophobic interactions in which the –Cl (chloro) substituent of imidazo[1,2-a] pyridine moiety formed three (3) alkyl and pi-alkyl hydrophobic with different residues (Leu 58, Ala 118, and Phe 121), and its delocalized Π-electrons formed amide-pi stacked and pi-alkyl interaction with Leu 58 and Pro 221 respectively. The bromo substituent (−Br) attached to the benzene formed four (4) pi-alkyl interaction with Ile 100, Phe 69, Ala 97, and Pro 167 residues while delocalized Π-electron in the benzene formed a pi-pi T-shaped interaction, pi-sigma, and pi-alkyl interactions with Phe 69, Leu 65, and Leu 166 accordingly. Other interactions comprise one (1) C–H bond with His 114 and two (2) pi-anion electrostatic interactions with Glu 159 as in Fig. 7a.

**Fig. 8:**
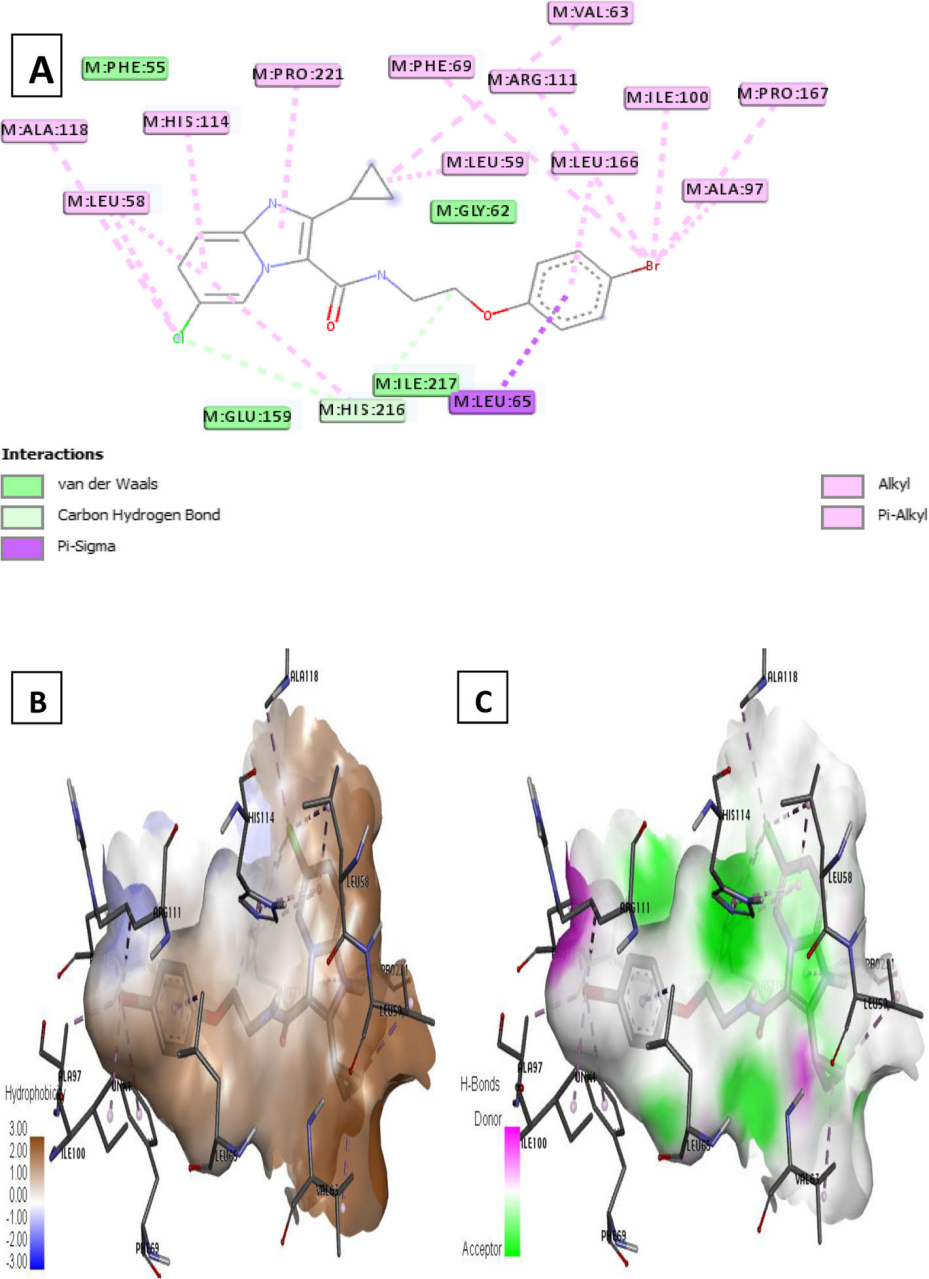
Docking interaction of complex 22. **a** 2D docked view of complex 22. **b** Hydrophobicity surfaces of complex 22. **c** Hydrogen bond surfaces of complex 22

Ligand 22 formed two (2) C–H bond interactions with the residue His 159 and one (1) van der Waals interaction with Ile 217. Also, ligand 22 formed fifteen (15) hydrophobic interactions which encompass pi-sigma with Leu 65 while pi-alkyl and alkyl hydrophobic involved Ala 97, Pro 167, Ile 100, Leu 166, Arg 111, Leu 59, Val 63, Phe 69, Pro 221, His 114, Leu 58, Ala 118, and Leu 65 at different interaction distances as elucidated in Fig. 8a.

**Fig. 9:**
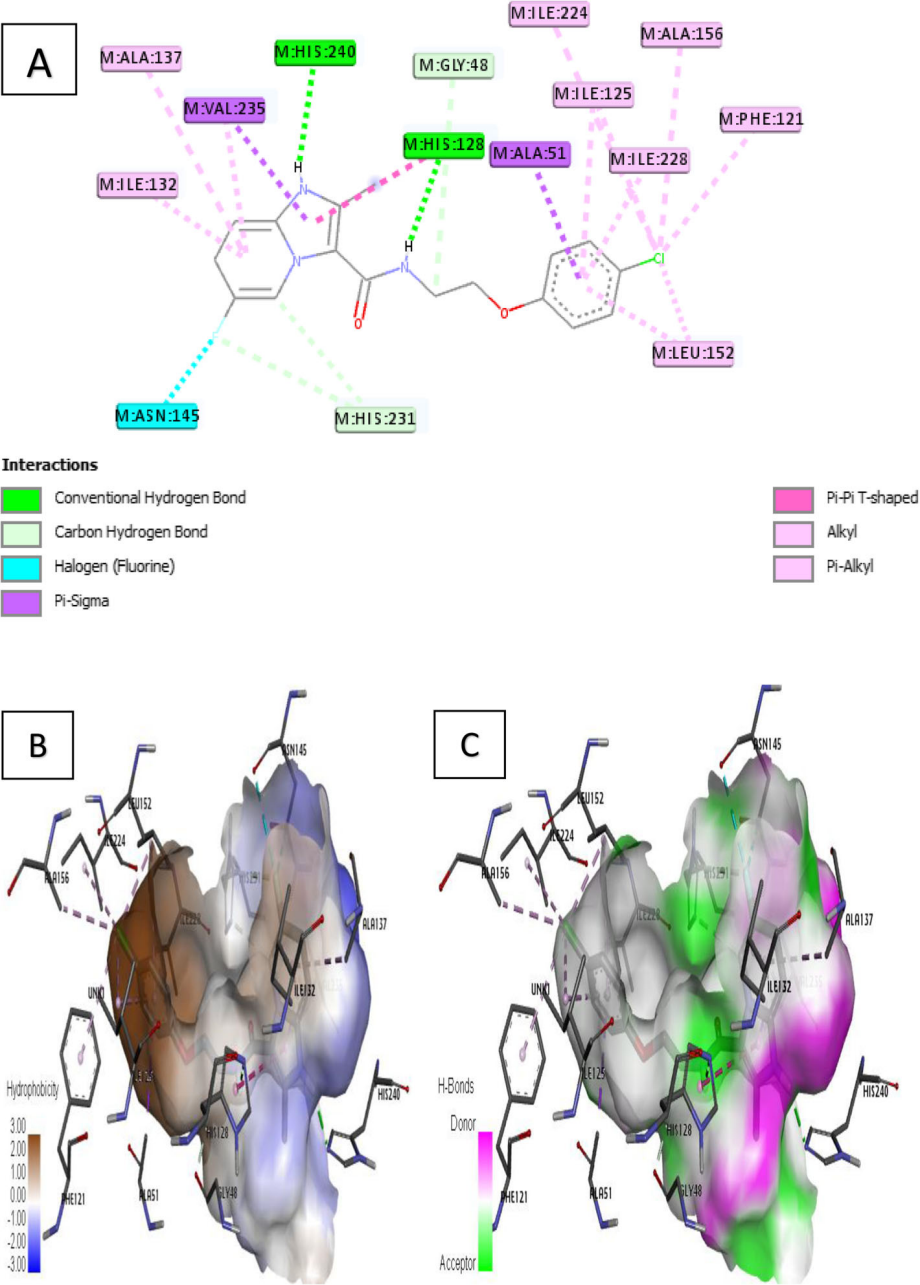
Docking interaction of complex 26. **a** 2D docked view of complex 26. **b** Hydrophobicity surfaces of complex 26. **c** Hydrogen bond surfaces of complex 26

Ligand 26 formed two (2) conventional H-bonds where –NH of imidazo[1,2-a] pyridine fragment interacts with His 240 and also –NH of acetamide fragment interacts with His 128 different distances respectively. In addition, ligand 26 also formed fourteen (14) hydrophobic interaction including pi-pi T-shaped, pi-sigma, pi-alkyl, and alkyl interaction types with residues such as Leu 152, Phe 121, Ile 228, Ala 156, Ile 125, Ile 224, Ala 51, His 128, Val 235, Ala 137, and Ile 132. Other interactions include three (3) C–H bonds with His 231, Gly 48, and a halogen interaction involving the fluoro substituent and Asn 145 amino acid residue as shown in Fig. 9a.

**Fig. 10:**
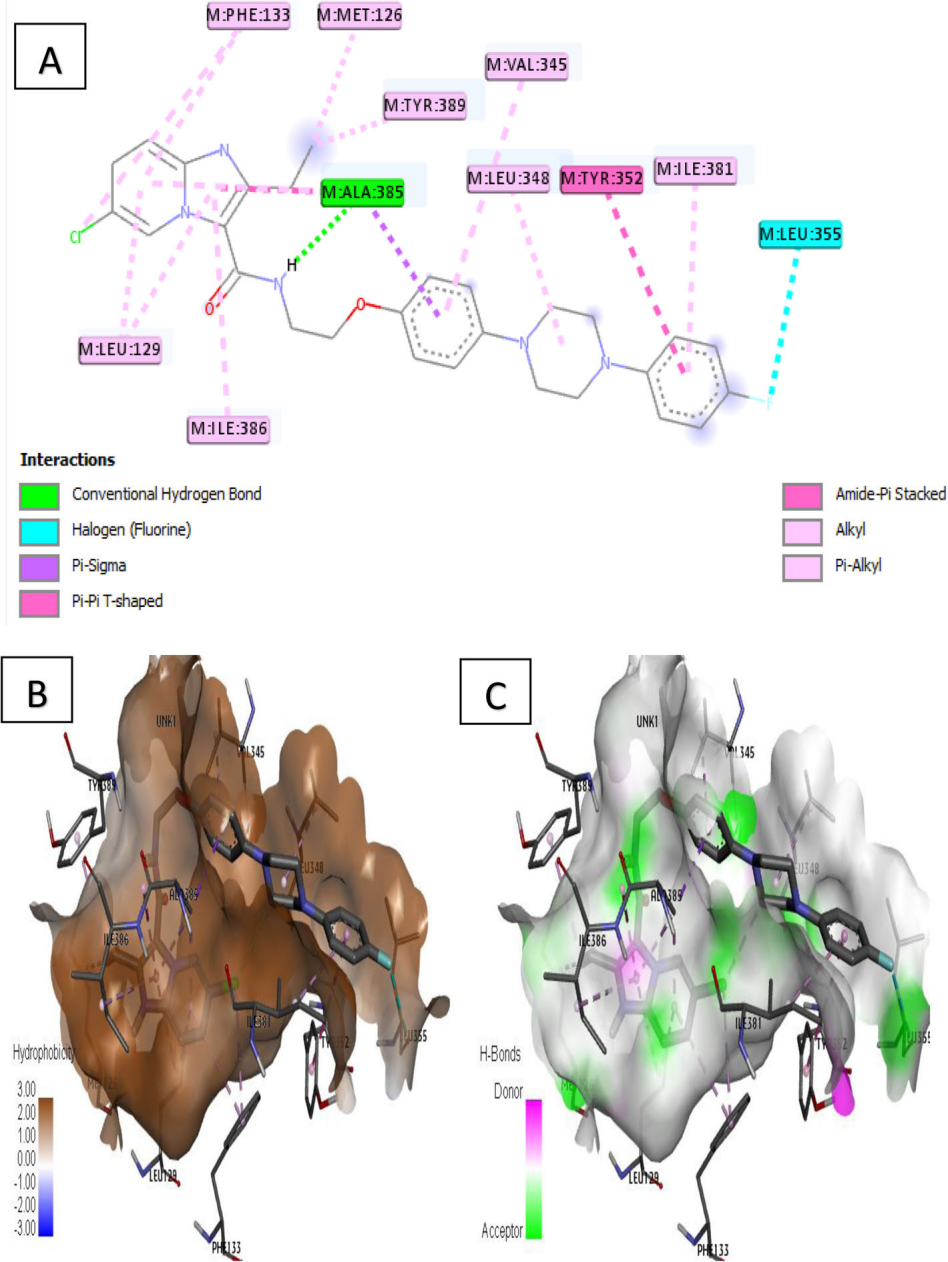
Docking interaction of complex 35. **a** 2D docked view of complex 35. **b** Hydrophobicity surfaces of complex 35. **c** Hydrogen bond surfaces of complex 35

Ligand 35 formed one (1) conventional H-bond due to the residual interaction between –NH of acetamide fragment and Ala 385 amino acid. Furthermore, ligand 35 also formed thirteen (13) hydrophobic interactions including pi-pi T-shaped, amide-pi-stacked, pi-sigma, pi-alkyl, and alkyl interaction types with residues such as Phe 133, Met 126, Leu 129, Ile 386, Try 389, Ala 385, Val 345, Leu 348, Tyr 352, and Ile 381. Halogen residual interaction was also formed between fluoro substituent of benzene and Leu 355 as elucidated in Fig. 10a.

Furthermore, receptor surfaces (hydrophobicity and hydrogen bonds) were created to have insight into the inner workings of the QcrB modeled receptor. The hydropathy index of the ligand protein was measured as the hydrophobicity surfaces which show the hydrophilic or hydrophobic properties of the amino acid side chains shown in Figs. 6b, 7b, 8b, 9b, and 10b. In addition, the hydrophobicity of the amino acid residue surfaces are colored from blue for hydrophilic to brown for hydrophobic accordingly. For H-bond surfaces, H-bond donors are colored as magenta surfaces while H-bond acceptors are colored in green as shown in Figs. 6c, 7c, 8c, 9c, and 10c. The binding modes of the docking output (Figs. 6, 7, 8, 9, and 10) revealed a high amount of hydrophobic/π interactions such as π–alkyl, π–sigma, and π–π type of interactions in conjunction with the presence of some hydrogen bonds and other noticeable interactions that contributed to the ligand-protein complex stability in the binding pockets of the modeled protein.

## Conclusion

*M. tuberculosis* QcrB is a potential molecular target that can be utilized in the development and design of new anti-tubercular compounds to fight the world’s TB menace. Research in this direction must continue to save the human race against the deadly disease. Thus, this research gives a firsthand theoretical insight on the prediction of binding affinities as well as the binding modes of some compounds of N-(2-phenoxy) ethyl imidazo[1,2-a] pyridine-3-carboxamide (IPA) with homology-modeled *M. tuberculosis* QcrB. The docking output revealed good binding affinity values ranging from − 6.5 to − 10.1 kcal/mol which confirms their resilience potency. Furthermore, ligands 2, 7, 22, 26, and 35 scored higher binding affinity values of − 9.60, − 9.80, − 10.10, − 10.00, and − 10.00 kcal/mol respectively. This is an indication that the ligands possess better conformation with the active site of the modeled protein when compared with other ligands of the data set and standard drug isoniazid (− 6.0 kcal/mol). The research outcomes set a course for structure-based drug design and revealed some potential hits for future discovery of more hypothetical anti-tubercular drugs. Besides, the study encouraged further validation of the predicted protein target through experimental research, characterization, and applications.

## Data Availability

Data sharing is not applicable to this article as no datasets were generated or analyzed during the current study
